# Supplementary material for: Natriuretic Peptide Expression and Function in GH3 Somatolactotropes and Feline Somatotrope Pituitary Tumours
Source: Int J Mol Sci. 2021 Jan 22;22(3):1076. doi: 10.3390/ijms22031076 (PMC7865297; doi:10.3390/ijms22031076)
Supplement: Supplementary file 1 [file ijms-22-01076-s001.pdf]

Supplemental Table S1 Primer sequences for multiplex RT-qPCR assays

Primer Name	Product size	Accession Number	Left sequence	Right sequence
Cat GHR	100	XM_011286348	TGGATATTGATGACCCCGAT	TCCTTTGCTCCAAGGATGTT
Cat NPPA	108	XM_003989498	GTCAGCTCTTGTGGCAAACA	AAGGCATCTTGTCCTCCAAA
Cat NPR1	116	XM_003999769	TCCAGGATGGAGTCTAACGG	TTCATTTCTACGTCCCCTCG
Cat DRD2	123	XM_003992361	ATCCACTGAACCTGTCCTGG	AGGTGAGAAGCGTGGCATAG
Cat NPPC	131	XM_003991256	TGCTCACGCTACTCTCGCT	TTTGTCGCCCTTCTTCTGAC
Cat NPR2	138	XM_003995612	CTTTGACTTGGACGACCCAT	TCCTTCTCCAGCATCAGCTT
Cat SSTR3	145	XM_003989238	ACGTGCTCAACATCGTCAAC	TGCTTGAAGCGGTAGGAGAG
Cat SSTR1	152	XM_011283202	CCTTTCGGACAACTTCAAGC	CAGACTCCAGGTTCTCTGGC
Cat GHSR	159	XM_003991905	CCTGCTCTGCAAACTCTTCC	ACCAGGATGACCAGCTTCAC
Cat SSTR5	166	XM_006942579	ATCTGCCTGTGCTACCTGCT	GGTTGACGATGTTGACGATG
Cat NPR3	173	XM_006928064	ACGAAACCAGAATCGTGGAG	CTGGCTTCTCCTCTCAATGG
Cat RPL18	180	XM_003997532	GGATGATCCGGAAGATGAAG	GGTCGAAGGTGAGGATCTTG
Cat SSTR4	187	XM_003983838	AGACGGCCACCAACATCTAC	CTGAGCACCGTCAGACAGAA
Cat NPPB	194	NM_001009244	AGGGGCAACGATCTCTTTCT	AGCTCTGAAACTGTGTCCCG
Cat SSTR2	201	NM_001309046	GATCGATACCTGGCTGTGGT	GTACCATGCCCCAGATTCAC
Kan(r)	288	N/A	ATCATCAGCATTGCATTCGATTCCT GTTTG	ATTCCGACTCGTCCAACATC
Nppa	137	NM_012612	CCGATAGATCTGCCCTCTTG	CCCCCATCCTAACTGTCCTA
ActB	144	NM_007393	GTACCACCATGTACCCAGGC	GAGGAGGACTCGCGTTCATG
Npr2	151	NM_173788	ATGTCTCCTCGACCACCAAG	CGCTTGAATGACCGAGAACC
Npr1	158	NM_008727	CTTGGAATTCCTGAAGCAGC	CCGAGGACGAGATACAGGTC
Nppb	165	NM_031545	GCAGAAGATAGACCGGATCG	CGAGAAGAAAGGGGTCGAGA
Npr3	172	NM_012868	CGGGACGATTCGTACTTGTT	AGGTCCACCGGATACTTCTG
Nppc	186	NM_053750	GGTCATCCTTGGTCATCAGC	CTCCCCTCCCCTACTAAAAT
Aip	193	NM_172327	GGCATAGGTGAGGAGAGCTG	TTTCACTTCCCCGAGGTCAA
Egr1	221	NM_012551	CAGGAGTGATGAACGCAAGA	GTCACGGGTGGAGAATGAGG
cFos	230	NM_022197	GGGAGCTGACAGATACGCTC	AGACACTGGAGGGACCTGAA
Prl	238	NM_012629	TCTGTTCTGCCAAAATGTGC	TAGTTACTGACGGGGTGA
Pou1f1	151	NM_013008	TTTATCCAAGTGGCTGGAGG	TCCTTAGCGGCGATACTGAT
Insr	158	NM_017071	AGGGTGAGGTAGAGACCCGT	ACCACGTGATGACAGGTGAA
Sstr5	165	NM_012882	GTCAACCTGGCCTTCACACT	AGTTGTCCGAGAGAAA
